# POWRS: Position-Sensitive Motif Discovery

**DOI:** 10.1371/journal.pone.0040373

**Published:** 2012-07-05

**Authors:** Ian W. Davis, Christopher Benninger, Philip N. Benfey, Tedd Elich

**Affiliations:** 1 GrassRoots Biotechnology, Durham, North Carolina, United States of America; 2 Department of Biology, Duke University, Durham, North Carolina, United States of America; 3 Center for Systems Biology, Duke University, Durham, North Carolina, United States of America; Lawrence Berkeley National Laboratory, United States of America

## Abstract

Transcription factors and the short, often degenerate DNA sequences they recognize are central regulators of gene expression, but their regulatory code is challenging to dissect experimentally. Thus, computational approaches have long been used to identify putative regulatory elements from the patterns in promoter sequences. Here we present a new algorithm “POWRS” (POsition-sensitive WoRd Set) for identifying regulatory sequence motifs, specifically developed to address two common shortcomings of existing algorithms. First, POWRS uses the position-specific enrichment of regulatory elements near transcription start sites to significantly increase sensitivity, while providing new information about the preferred localization of those elements. Second, POWRS forgoes position weight matrices for a discrete motif representation that appears more resistant to over-generalization. We apply this algorithm to discover sequences related to constitutive, high-level gene expression in the model plant Arabidopsis thaliana, and then experimentally validate the importance of those elements by systematically mutating two endogenous promoters and measuring the effect on gene expression levels. This provides a foundation for future efforts to rationally engineer gene expression in plants, a problem of great importance in developing biotech crop varieties. Availability: BSD-licensed Python code at http://grassrootsbio.com/papers/powrs/.

## Introduction

The binding of transcription factors (TFs) to specific DNA binding sites (TFBS) is a key mechanism in the regulation of gene expression. Although genome-wide binding data for some TFs is becoming available, there are still many cases in which the TF(s) responsible for a regulatory program are unknown. Thus, computational prediction of TFBS motifs is a long-standing problem in bioinformatics. Motifs are traditionally identified from promoters of co-regulated genes, but the same algorithms apply to ChIP-chip and ChIP-seq data, and to miRNA binding sites in 3′ UTRs.

Scores of motif identification programs have been developed, spanning a wide range of approaches and problem niches. Das and Dai provide a comprehensive review of motif finders [1], so below we touch on several of the issues that differentiate them.

To begin, motif finders differ in the input data they are tailored for: few sequences or many; positive sequences only, positives and negatives, or a continuum of scored sequences. TreeMotif is a recent example of an algorithm tailored to extracting long, degenerate motifs from a few tens of sequences that all share a common TFBS [2]. By contrast, many algorithms deal with genomic-scale data. Most, like Amadeus [3], expect sequences to be classified as “positive” or “negative”, with most positive sequences expected to share common TFBS motif(s). Others, like cERMIT [4], associate a score with each sequence that reflects how likely it is to belong to the positive group.

Motif finders also differ in the ways they search for and evaluate putative motifs. Probabilistic methods [5], [6] and enumeration of short words [3], [4] are two of the most popular search strategies. Many algorithms include greedy, stochastic, or heuristic components, as global optimization is generally not feasible. Putative motifs have been scored using the binomial and hypergeometric distributions [3], mutual information [7], various types of Z-score [4], [8], Bayesian priors [9], and many other approaches. In order to compute these statistics, many algorithms also compute a background model. This is often a Hidden Markov model (HMM), and can be done from positive sequences only if necessary. Other “discriminative” algorithms forego a background model and directly discriminate between real positive and negative sequences [8].

As a consequence of the above considerations, motif finders represent the TFBS they detect in different ways. In order of increasing complexity, the major representations are a single consensus sequence, a degenerate consensus sequence using IUPAC codes, and a position weight matrix (PWM). Although PWMs are most popular, they have limitations. Directly optimizing a PWM is computationally difficult and often gives limited sensitivity [1]. On the other hand, clustering simpler results into a PWM during post-processing is also hard: our experience is that over-zealous clustering often merges biologically distinct motifs, yielding uninformative PWMs. Note that IUPAC codes are just PWMs constrained to have non-zero weights equal in each column, and so have similar problems.

Finally, motif finders pay more or less attention to the location of motifs. Most algorithms assume that TFBS motifs are distributed uniformly along the lengths of the sequences, and limit analysis to an arbitrary number of bases upstream of the transcription start site (TSS). However, recent TSS mapping plus ChIP in Drosophila [10] shows TFBS positions often have sharp positional constraints around the TSS. As far back as 2004, FitzGerald et al. [11] observed that seven common human TFBS cluster strongly near the TSS, and similar patterns have been observed more recently in plants [12]. Genome-wide analysis also detects preferred motif pair spacings [13]. Although a few motif finders post-process results to look for position bias [3], [7], it has not historically been the focus of the motif discovery process. (The position-conscious NCBI program A-GLAM was a notable early exception [14], [15], as are several recent algorithms; see Discussion.) By contrast, we expect and observe a continuous gradient of TFBS density that peaks near the TSS. We exploit this observation to increase the sensitivity of our algorithm, because motifs that are informative near the TSS (where TF binding impacts transcription) can be uninformative far from the TSS (where TF binding may not have functional consequences). Similar considerations arise from the strand specificity of TFs.

Our motivation is to discover novel, functional regulatory motifs in plant species (whose regulatory mechanisms are less characterized than e.g. yeast or human), and to use those motifs in engineering transgene expression. To that end, we aim to overcome two limitations of existing approaches. First, most motif finders ignore position and strand effects; we characterize the position- and strand-specificity of any discovered motif. Second, PWMs are often problematic in motif finding. Instead, we represent motifs as a single, non-degenerate consensus sequence, plus some variants that differ by a single mutation. This captures a limited but useful amount of degeneracy without the number of free parameters (and accompanying computational cost) of a full PWM. Identifying the consensus (and hopefully optimal) sequence, strand, and position for each TFBS is particularly relevant for engineering gene expression, but is also important for a general understanding of transcriptional regulatory systems.

In this work, we describe the POWRS (POsition-sensitive WoRd Set) motif identification algorithm. We compare its performance to well-known and state-of-the-art algorithms on a benchmark set of TF and miRNA data, and demonstrate the advantages of our motif model and of position- and strand-specific search relative to an identical algorithm without those features. We then apply our algorithm to motif discovery in a set of co-regulated genes from the model plant *Arabidopsis thaliana*, and validate our motif predictions by systematically mutating all 10-bp segments of two endogenous promoters.

## Materials and Methods

### POWRS

POWRS starts with two sets of sequences, both aligned on a common feature (the TSS of promoters or the 5′ ends of 3′ UTRs in the examples here). The first set of sequences (typically a few hundred) are hypothesized to share one or more motifs, while the second set (typically the rest of the genome) provide a background distribution. Using p-values from the binomial distribution, POWRS finds the words that are most enriched in the first set of sequences, and for each word, the region of these sequences in which the enrichment is most significant. Closely related words are then greedily grouped together to form motifs.

Formally, let *A* be the set of sequences of interest and *B* be the set of background sequences. If *k* is a short word of specified length, let variants *k* be all words of the same length that differ from *k* by at most one mutation. Let motif *K* be a set of words consisting of seed k* and zero or more of variants k*. Then 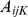 is the set of all sequences 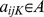 that contain at least one word 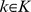 in the coordinate range 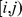, and 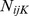 is the set of all sequences 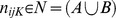 that contain at least one word 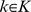 in the coordinate range 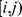. We define the following scores based on the binomial distribution (*x* successes, *y* trials, probability of success *p*):
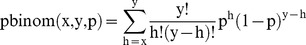


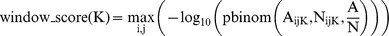



For each possible seed word k* of a given length, POWRS then finds its top-scoring motif *K* and the motif’s region of maximal enrichment 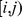:

for each possible seed word k* of a given length:

initialize 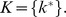


repeat:
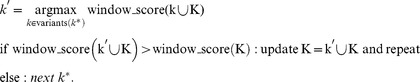



For each motif considered, we calculate the continuous window of sequence positions over which the highest score is achieved (for example, from −300 to −75 relative to the TSS). Shorter windows trade off potentially higher levels of enrichment against lower total counts, and hence lower statistical significance. The scoring is O(n^2^) in the length of the sequence, but by choosing an appropriate granularity for *i* and *j* (e.g. 25 bp for 1000 bp sequences), the total number of windows to be tested can be kept small. That is, with 25 bp granularity, the optimal window found could be −300 to −75 relative to the TSS, but not −298 to −86. By sorting match positions of each word in each sequence, we can efficiently fill a 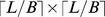 table of counts for the binomial test (L is maximum sequence length, B is the search granularity; row denotes window start position *i*, column denotes window end position *j*; upper triangle only). Scores (p-values) are not directly corrected for multiple testing, because the tests of overlapping windows and similar motifs are not independent. Corrected p-values can be obtained by permutation or bootstrapping, at additional computational cost. As a rule of thumb, scores below 10 (i.e. P>10^−10^) are unlikely to be statistically significant.

Each seed word is then made into a motif by adding close variant words to the motif definition, in each cycle greedily adding the variant that most increases the score. Variant words differ from the seed word at exactly one position (one mutation). We call this motif model a “word set” to distinguish it from a PWM motif model. Sequences are counted for scoring purposes if they match at least one of the words in the motif, in the appropriate window. In addition to re-assessing the optimal window at each cycle (as above), motifs are tested against the sense strand only and against both strands to see which gives the higher score. Iteration stops when no variant can be added that will improve the score. Note that only variants of the original seed motif are considered for inclusion, not variants of variants, although every possible word gets an opportunity to be a seed.

Each seed word of length 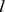 has 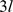 variants, and so optimizing the motif requires 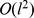 cycles of window optimization in the worst case. Although this proceeds quickly, in the interest of saving compute time we generally choose to optimize only the most promising words from the initial pass. In the results here, we optimized motifs for the 800 single words (i.e., k*) with the most significant initial p-values. We used granularities of 50 bp on the human TFs (2000 bp sequence, GRCh37), 500 bp on the 3′ UTRs (5000 bp sequence, GRCh37), and 25 bp on the Arabidopsis promoters (1050 bp sequence, TAIR9). We also employ a suffix array to speed motif searching. Run times for the TF and miRNA data sets were 20–25 minutes per TF/miRNA on a single Xeon processor.

Results of POWRS deliberately include similar, “redundant” results for each motif. We do automatically suppress any motif whose seed is used as a variant of a higher-scoring motif, which already sharply limits the number of duplicates. For ranking results in the benchmark, we also group together motifs that obviously refer to the same TFBS, i.e. those that have at least 6 contiguous identical bases between their seeds, allowing for shifts. These generally arise because the full motif is longer than 8 bp, but occasionally show variants that differ from the consensus in 2 of 8 positions.

### Promoter Analysis

Arabidopsis genes AT1G22840 (GR2A) and AT3G62250 (GR11A) were identified as highly expressed constitutive genes as described in Results. Because both genes had very short 5′ intergenic regions (195 bp and 141 bp, respectively), we hypothesized that their promoters might be compact and ideal for functional dissection. The intergenic regions, as well as 10 bp sequential transversion series thereof, were generated by synthesis (Genscript), cloned in front of a GFP reporter in a binary expression vector (Cambia), and transformed into Arabidopsis thaliana Col-0 by the floral dip method [16]. GFP expression in root tissue was determined by quantitative RT- PCR on four or five low-copy non-segregating T3 lines from each transversion series member. Approximately 50 seedlings per line were grown on standard MS media in 100 mm square plates for seven days at 23°C, 16 hours light, 8 hours dark, and then pooled together for harvesting. Root tissue was homogenized in liquid nitrogen via bead milling and total RNA and genomic DNA was extracted using the Allprep DNA/RNA kit (Qiagen). cDNA was generated from total RNA using Superscript VILO cDNA synthesis kit (Invitrogen) per the manufacturer’s instructions. Multiplex qPCR TaqMan assays were conducted on cDNA and genomic DNA using the CFX96 Real-Time PCR Detection System (Bio-Rad) with primers and probes specific for GFP, PDS1, and the “housekeeping” gene UBC9, as follows: GFP primer forward –5′CGTGCAGGAGAGGACCAT; GFP primer reverse –5′TGTCTCCCTCAAACTTGACTTCAG; GFP probe –5′/56-FAM/AGTTCCCGT/ZEN/CGTCCTTGAAGAAG/3IABkFQ; UBC9primer forward –5′ATGGAAGCATCTGCCTCGACATCT; UBC9 primer reverse –5′AGGATCATCTGGGTTTGGATCCGT; UBC9probe –5′/5TEX615/AGCAGTGGAGTCCTGCTCTCACAATT/3IAbRQSp; PDS1 primer forward –5′TCACGGCTCTTGTCGTTCCTTCTT; PDS1 primer reverse –5′TGGAGAAAGCTGACTCTGCGTCTT; PDS1 probe –5′/5TEX615/TCGGTGTTAGAGCCGTTGCGATTGAA/3IAbRQSp. The amplification protocol was 95°C for 9 min, followed by 40 repeats of 95°C for 15 s, 57°C for 30 s, 72°C for 30 s, read.

Three technical qRT-PCR replicates were performed on each biological replicate. Data were processed using CFX Manager software (Bio-Rad). To determine relative GFP expression level and copy number, PCR reaction efficiency was calculated using LinRegPCR software (Ruijter). Ct and baseline threshold values were obtained via the CFX Manager software. Data analysis was then performed using the GenEx software package (MultiD Analyses AB). After correcting for reaction efficiency, the Ct values of technical replicates were averaged and the relative GFP expression and copy number was calculated by normalizing relative levels of *GFP* to *UBC9* in cDNA and *GFP* to *PDS1* in genomic DNA, respectively. GFP expression in individual lines was normalized to copy number and expressed relative to a CaMV 35S control.

## Results

We first compare the performance of POWRS to other motif finders on benchmark sets of transcription factors and miRNAs from the literature, finding POWRS to be as successful as the best existing algorithms and to provide additional data about motif positioning. Furthermore, we show that position-specificity and the word-set motif model contribute to POWRS’s success in finding motifs. Finally, we apply POWRS to *de novo* motif discovery in the model plant *Arabidopsis thaliana*. We then show that mutating the identified motifs compromises activity of two endogenous promoters, but mutating other regions generally does not.

### TFBS Benchmark

Table 1 compares the performance of POWRS and its variations to 7 other motif finders on a set of 9 human TFs, using the procedures and results reported by Linhart et al. [3] and Keilwagen et al. [17]. POWRS performs equivalently to the other two best programs, Amadeus and Weeder, succeeding and failing on the same targets. However, POWRS provides additional information about the position and strand specificity of each motif (Table 2). Although Amadeus searches for strand and location bias, among these targets it only detects localization of CREB, which it places at −60. Dispom also performed well and does calculate motif positions, but these were not reported [17]. Interestingly, no TF’s optimal window extended beyond −600. Typical practice is to use 1–3 kb of promoter sequence, but this result suggests using only ∼500 bp may result in higher success rates for motif discovery in co-regulated gene sets.

**Table 1:** Comparison of motif finders on benchmark and *de novo* discovery data sets.

Group	Target	Ref.	POWRS	POWRS-FL	Simple8	Simple6	Amadeus	Weeder	Trawler	YMF	AlignACE	MEME	Dispom
**TFs**	CREB	MA0018.2	2	3	2	2	2	X(2)	X	X	X	X	X(2)
	E2F	MA0024.1	2	2	X	1	1	X(1)	2	X	X	X	1
	ETS1	MA0098.1	2	1	1	1	3	1	X	X(1)	X	X	2
	HNF1a	MA0046.1	1	X	X	2	3(1)	X	1	X	X(4)	X	2
	NFkB1	MA0105.1	1	X	X	X	1	X(4)	2	X	X	X	2
	P53	MA106.1	X	X	X	X	1(X)	3	X	X	X	X	X
	Sox2	MA0143.1	X	X	X	X	X	X	X	X	X	X	X
	SRF	MA0083.1	1	1	X	1	1	2(1)	2	4	X	X	1
	YY1	MA0095.1	1	2	X	1	1	1	1	1	1	X	1
**miRNAs**	let7 (B)	MIMAT0000062	X	X	X	X	X	X	X	X	X	X	nd
	let7 (J)	MIMAT0000062	1	1	X	X	1	1	1	X	X	X	nd
	miR106b	MIMAT0000680	1	1	1	1	1	1	1	2	X	1	nd
	miR124	MIMAT0000422	1	1	1	1	1	1	1	1	X	1	nd
	miR16	MIMAT0000069	1	1	1	1	1	1	1	X	X	1	nd
	miR1	MIMAT0000416	1	1	1	1	1	1	2	X	X	1	nd
	miR34 (C)	MIMAT0000686	X	X	X	X	X	X	X	X(4)	X	X	nd
	miR34 (H)	MIMAT0000686	1	1	1	1	1	1	2	X	X	2	nd
	miR373	MIMAT0000726	1	1	1	1	1	1	2(X)	1	X	1	nd
**Arabid.**	telo box	AAACCCTAGC	1	2	1	2	nd	nd	nd	nd	nd	nd	nd
	Site II	AAGGCCCAWT	2	1	2	1	nd	nd	nd	nd	nd	nd	nd
	TATA box	TCTATAAAA	3	X	X	X	nd	nd	nd	nd	nd	nd	nd

Rank of the “correct” motif in the output of various programs. “Target” refers to data sets defined in [3]. “Ref.” gives the accession number in the JASPAR or miRbase database, or the target consensus sequence. X, no match in the top 4 results; nd, not determined (i.e. the tools were not run due to licensing restrictions on non-academic use). Results for Amadeus, Weeder, Trawler, YMF, AlignACE, and MEME are quoted from [3], as several are not freely available outside academia. Results for Dispom are quoted from [17]. “POWRS-FL” is POWRS without position sensitivity (“full length”). “Simple8” and “Simple6” are the whole-sequence, binomial-scoring algorithm described in the text, using 8-mers and 6-mers respectively. A result was considered correct if at least 6 contiguous bases of the result matched the literature motif (except ETS1 and YY1, which are effectively 4 bases long). The ranking from the more permissive PWM-based metric in [3] is shown in parenthesis where it disagrees.

**Table 2:** Detailed results of POWRS motif searches.

Target	Ref.	Rank	Score	Motif	Start	End	Strands?
**CREB**	TGACGTNW	2	45.4	[Act][Gat][Tg][Gc]ACG[Tac]	−400	0	Both
**E2F**	TTTSSCGC	2	10.7	TT[Ga][Gt]C[Ga]C[Gc]	−450	−50	Both
**ETS1**	NWTCCN	2	50.8	[Cagt]A[Cagt]TTCCG	−550	0	Both
**HNF1a**	GGTTAATNWTTNNN	1	15.2	TTA[Ac][Tc][Gac]A[Tcg]	−250	0	Both
**NFkB1**	GGGGRWYYCCC	1	11.4	G[Gt][Ag][At][At][Tac][Cat]C	−400	0	Both
**P53**	NNRRRCATGYCCGGGCATGT	–	–	–	–	–	–
**Sox2**	CCWTTGTNNTNNNNN	–	–	–	–	–	–
**SRF**	GCCCWTATAWGG	1	12.8	C[Ca][At][Ta][Agt]T[At][Ta]	−300	0	One
**YY1**	NCCATN	1	67.2	GC[Cg]AT[Gact]T[Tc]	−350	0	Both
**let7 (B)**	CTACCTCA	–	–	–	–	–	–
**let7 (J)**	CTACCTCA	1	24.3	[Ca]TA[Cag]CT[Cg][Ta]	0	5000	One
**miR106b**	GCACTTTA	1	29.1	GCACTTT[Act]	0	4000	One
**miR124**	GTGCCTTA	1	31.9	[Gt]TGCCTT[Acgt]	0	5000	One
**miR16**	TGCTGCTA	1	31.3	[Tac]GCTGCT[Agt]	0	3500	One
**miR1**	ACATTCCA	1	23.8	[Agt]CATTCC[Agt]	0	2000	One
**miR34 (C)**	CACTGCCT	–	–	–	–	–	–
**miR34 (H)**	CACTGCCT	1	45.9	[Cagt]AC[Tag]GCC[Tag]	0	2000	One
**miR373**	AGCACTTC	1	18.4	[Tac]A[Ag]GCACT	0	1000	One
**telo box**	AAACCCTAGC	1	28.0	[Ag]C[Ct]C[Ta][At][Gat][Tacg]	−75	+25	Both
**Site II**	AAGGCCCAWT	2	23.4	[Agt][Gat][Ga]CC[Cg]A[Acgt]	−150	−25	One
**TATA box**	TCTATAAAA	3	17.2	[Tacg][Ca][Tg]ATAA[Ag]	−50	−25	One

“Target” refers to the data sets from [3]. Reference motifs are IUPAC approximations of PWMs from JASPAR (human TFs), seed sequences from miRbase (human miRNAs), or manual consensus sequences (Arabidopsis). (See Table S2 for the same data with PWMs from JASPAR shown as sequence logos.) Motifs are represented with the primary bases in uppercase and the variant bases in lowercase, with degenerate positions grouped in square brackets. Matching words are those that use at most one variant base, so [Tac]GCTGCT[Agt] = {TGCTGCTA, aGCTGCTA, cGCTGCTA, TGCTGCTg, TGCTGCTt}.

We note that for the CREB target (JASPAR MA0018.2), the three top performing programs (Amadeus, Weeder, and POWRS) all identify the consensus CTTCCG/CGGAAG as their top hit, with the CREB motif second. That top hit matches JASPAR motif MA0076.1, Elk4. A cursory search of the literature reveals that Elk4 and CREB both interact with the kinase Erk during the development of B cells, and both regulate cell expansion [18], suggesting this result reflects biology rather than artifact. This type of result should be expected in higher eukaryotes, where complex transcriptional programs are regulated by many interacting factors.

Table 2 and Table S2 show POWRS’ detailed predictions. The pattern of degeneracy in the word set motifs in general agrees well with that in JASPAR PWMs. As expected, POWRS automatically determined that most TFs’ range extends all the way to the TSS (position 0) and covers both strands. SRF is the only TF predicted to have a strand preference, which is surprising because the literature PWM is palindromic. However, slightly lower-scoring results for SRF cover both strands, suggesting the asymmetry, if any, is slight.

The original comparison [3] used 29 TF datasets, but unfortunately was referenced to the TRANSFAC database [19], which is not freely available outside of academia. Since the target motifs were not reproduced in the original comparison, we had to limit our comparison to the 9 TFs that had motifs listed in the free database JASPAR [20]. JASPAR has the additional advantage that there is only one canonical motif for each TF, whereas TRANSFAC lists up to 16 different PWMs for some TFs in the benchmark set.

### miRNA Benchmark

Table 1 also compares the performance of POWRS to other motif finders on a set of 7 miRNAs, two of which have data from two different teams, again as reported by Linhart et al. [3]. No data was published for Dispom. As this benchmark was referenced to the public miRbase [21] database, we were able to test against the entire set. Amadeus, Weeder, and POWRS again performed identically. As expected, POWRS predicted all motifs to occur on the sense strand only, while most TFBS were predicted to occur on both strands. Unsurprisingly, POWRS detected no strong localization of miRNA binding sites relative to the start of the 3′ UTR.

### Effect of Position Sensitivity on Motif Discovery

To investigate the impact of position sensitivity on our ability to detect TFBS motifs, we performed three experiments. First, we ran POWRS with positional granularity equal to the full sequence length, thereby eliminating position sensitivity from the algorithm. Table 1 shows that this “full length” variant performs significantly worse than the position-sensitive version of POWRS.

Second, we compared the results of POWRS to an even simpler algorithm that uses the same binomial scoring function, but examines single words only (no word sets, no degeneracy) over the entire sequence length. Results are reported as “Simple8” (8-mers) and “Simple6” (6-mers) in Table 1. The results demonstrate that a very simple discriminative algorithm is still sufficient to resolve many of the benchmark cases, although in some cases only a shorter 6-bp core of the motif could be reliably identified. In Arabidopsis, the simpler algorithm detects the Site II and telo box motifs, but does not detect the TATA box, presumably because the TATA box is highly position specific. Comparing the POWRS-FL and Simple8 results suggests that the word-set motif model does improve sensitivity without over-generalizing the motifs. Overall, POWRS is able to find correct motifs in 17 cases, versus 10–14 cases for the simpler algorithms.

Third, we compared the results of POWRS on a set of 118 Arabidopsis constitutive gene promoters (see below), in one case aligning them on the TSS annotated in TAIR9 [22], and in the other aligning them on the most probable TSS estimated from the available collection of Arabidopsis ESTs, essentially as in Troukhan et al. [23]. The pipeline used for annotations at TAIR favors the most 5′ TSS rather than the most common TSS, so the EST-aligned sequences are expected to display tighter distributions of TFBS, even though the TSS is predicted solely from the 5′ ends of ESTs and not from any sequence motifs. As expected, when the EST-aligned sequences were used, the score of the top motif hit increased from 25.0 to 28.0, and the score of the TATA box motif increased from 13.3 to 17.2. These scores represent improvements of 3 and 4 orders of magnitude in the binomial p-values. The positional distribution of TATA box sequences also tightened from (−25 to +50) to (−50 to −25). This demonstrates that better TSS annotation can improve motif discovery, and indirectly demonstrates that position-specific algorithms exploit that information for higher sensitivity.

### Motif Discovery in Arabidopsis

Using the microarray data of Brady et al. [24] and Schmid et al. [25], we identified 118 genes that were highly expressed throughout Arabidopsis plants (Table S1). For each gene, we extracted 1000 bp upstream and 50 bp downstream of the TSS annotated in the TAIR9 genome release [22]. We then used POWRS to search for motifs involved in this expression pattern, leading to the identification of 3 putative motifs.

The first motif has consensus AAACCCTAGC, and occurs on either strand primarily between -75 and +25 relative to the TSS. It occurs in that region in 46 of the 118 genes, and additional genes contain near matches to the motif. The PLACE database [26] identifies this motif as “Up2” (AAACCCTA) or “telo box” (AAACCCTAA). Although this motif has previously been associated with up-regulation in axillary buds, root primordia, and cycling cells, to the best of our knowledge it has not previously been connected to constitutive high expression [27], [28].

**Figure 1:**
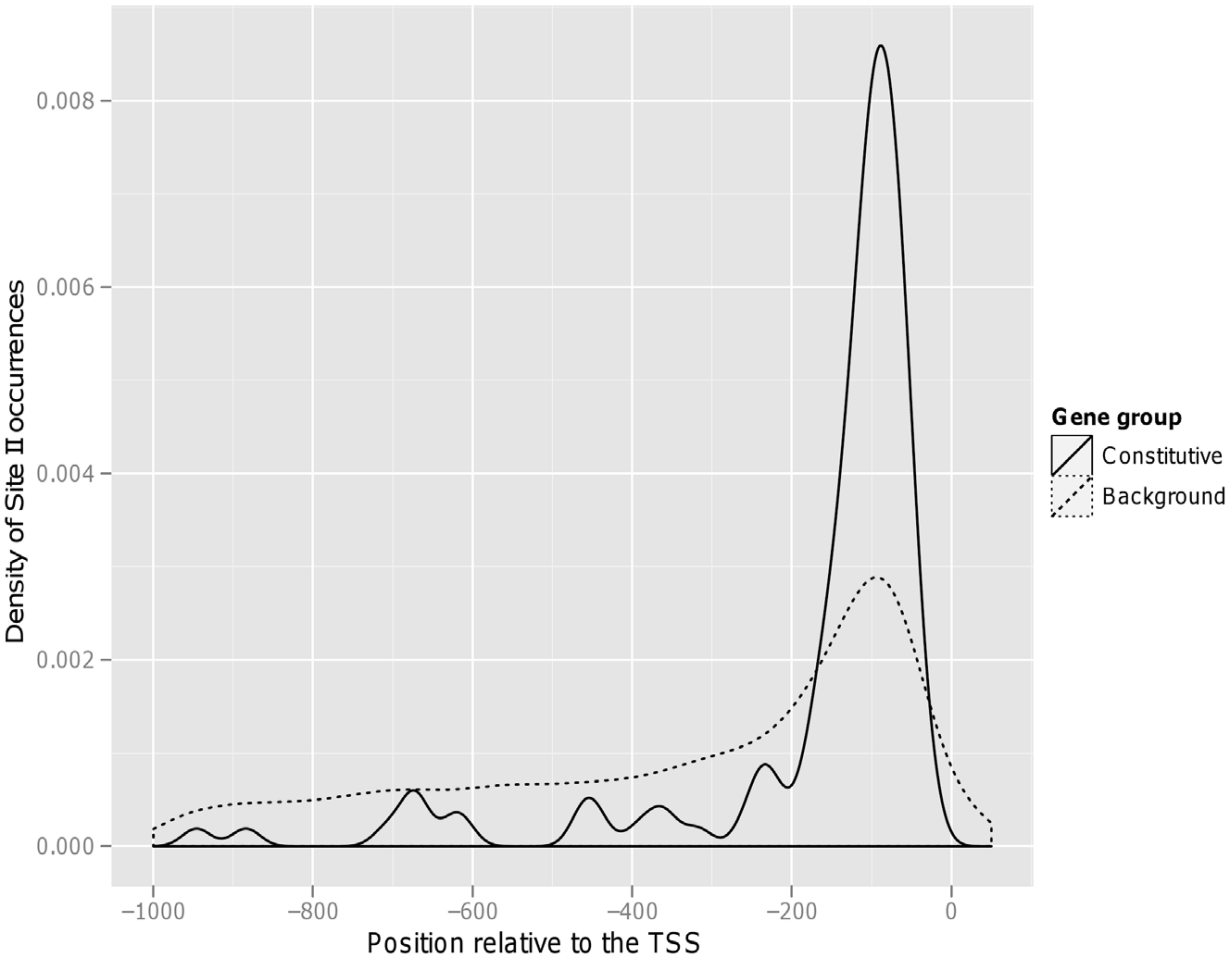
Graphical depiction of Site II motif matches in Arabidopsis. Smoothed histogram (kernel density estimate) of occurrences of the Site II motif in Arabidopsis promoters from the 118 constitutive genes of interest (solid line) or background genes (dashed line). The Site II motif is as defined in Table 2. Units of motif density are occurrences per base pair per sequence. POWRS reports maximal enrichment of Site II in the genes of interest relative to the background in the region from −150 to +25, in excellent agreement with what is seen here. Note that although Site II occurs more often near the TSS for all genes, the effect is significantly stronger among the genes of interest.

The second motif has consensus AAGGCCCAWT, and occurs preferentially on the sense strand between −150 and −25, in 47 of the 118 genes. PLACE identifies this motif as “Up1” (GGCCCAWWW) or “Site II element” (TGGGCY). The positional enrichment of this motif is depicted graphically in Figure 1. Again, this motif has been previously implicated in expression in cycling cells and meristems, but not constitutive high expression [27], [28].

The third motif has consensus TCTATAAAA, and occurs on the sense strand between −50 and −25, in 38 of the 118 genes; this is the TATA box. Although the literature reports that TATA boxes are more common in highly regulated genes than in “housekeeping” genes, we find strong enrichment of TATA boxes in these highly expressed constitutive genes, using a variety of different algorithms. That being said, we find the Site II and telo box motifs in both of the Arabidopsis promoters studied below, but neither contains a recognizable TATA box.

### Validation of Arabidopsis TFBS

**Figure 2:**
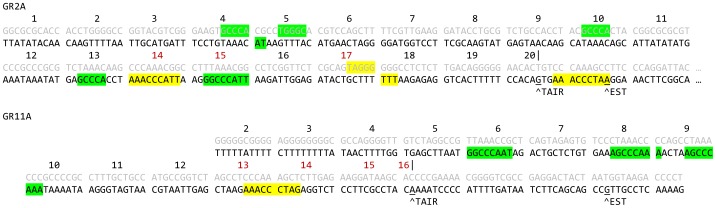
Transversion scheme in GR2A and GR11A. Endogenous sequence is shown in black, sequence after transversion is shown above in gray. Transcription starts sites annotated by TAIR9 [22] and inferred from EST data are indicated. Blocks for transversion are numbered and delimited by spaces. Natural Site II and telo box motifs are marked on the endogenous sequence in green and yellow respectively. Non-natural Site II and telo box motifs created by the transversions are marked on the transversion sequence; in some cases, these are split between natural and mutated sequences. Blocks whose transversion clearly disrupted promoter activity are numbered in red (compare to Figure 3).

**Figure 3:**
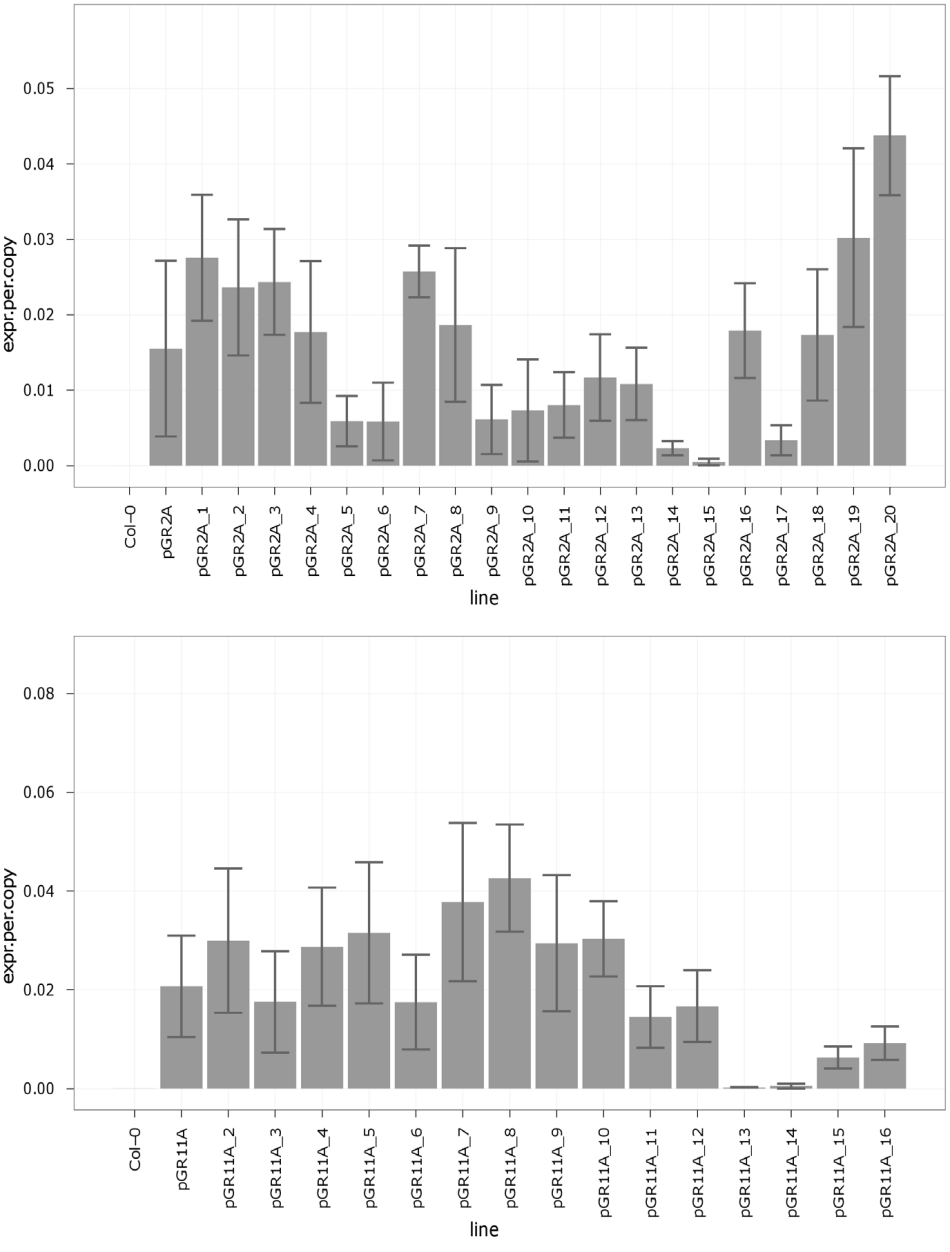
Transversion results for GR2A and GR11A. Mean and standard error of GFP expression driven by 10 bp transversion mutants of endogenous promoters GR2A and GR11A. Stable transgenic plants from 4–6 independent events per line were assayed by qRT-PCR and corrected for copy number.

To validate the impact of these motifs on gene expression, we systematically mutated two short, endogenous Arabidopsis promoters derived from the set of 118 strong, constitutive genes. Both promoters contain Site II and telo box motifs, though neither includes a recognizable TATA box. In each promoter, consecutive 10 bp segments were subjected to transversions (A↔C, G↔T), as diagrammed in Figure 2. Activity of the mutated promoters was compared to wild type by qRT-PCR (Figure 3). Although biological variation (presumably position effects) masks minor changes in activity, several 10 bp transversions clearly disrupted promoter activity: GR2A_14, 15, and 17; and GR11A_13, 14, 15, and 16. Most of these blocks substantially disrupt either a Site II or telo box motif. In GR2A, block 14 eliminates one of the two natural telo box motifs (the other is downstream of the annotated TSS and so unfortunately was not tested). Block 15 likewise eliminates the only complete Site II consensus sequence (block 13 contains the Site II core GCCCA only). Transversion of block 17, by contrast, actually introduces a new telo box motif, on the reverse strand and positioned between the two endogenous occurrences. This site may disrupt transcription through competition with the natural sites. In GR11A, there are three good matches to the full Site II motif (blocks 6, 8, and 9/10); disrupting any one of these individually may decrease expression slightly, but does not seriously compromise the promoter. Of course, not all motif matches will necessarily be biologically active TFBS (see Discussion); this may also explain part of the lack of response. On the other hand, there is only one telo box motif, split between blocks 13 and 14; both these transversions clearly disrupt activity. Finally, transversion of block 15 or 16 (which overlap by all but one base pair) also appears to disrupt promoter activity, but does not appear to involve any of the motifs identified here. In both promoters, a number of transversions show a modest and statistically insignificant decrease in activity (GR2A_5, 6, 9, 10, 11, 12, 13; GR11A_3, 6, 11, 12). We hypothesize this primarily reflects random biological variation, as the measurement error is sizable. Consistent with this hypothesis, only GR11A_6 contains a full length Site II or telo box motif.

## Discussion

We have described POWRS, a novel algorithm for discovering putative transcription factor binding motifs from sets of co-regulated genes. However, POWRS also determines the preferred location and strand for each motif, making it ideal for identifying likely active instances of a motif or for targeting genetic manipulations. In general, the motifs identified by POWRS in promoters of constitutive, highly expressed genes from Arabidopsis appear to be essential for the activity of those promoters. However, our mutagenesis experiments also show that other sequences can play a critical role in promoter activity, and that the effects of the motifs we identified can vary with position and number of copies. This is to be expected: the DNA motifs found by POWRS should correspond roughly to optimal TFBS on naked DNA, but not all instances of a motif will be biologically active, nor will all biologically relevant binding sites perfectly match the motif. Other researchers have shown that histone modifications, chromatin state, and DNA accessibility are all important for discriminating active from inactive sites; one example is the CENTIPEDE algorithm of Pique-Regi et al. [29].

In practice, discovery of motifs from co-regulated gene sets is a messy affair. When the selection is based on co-regulation or co-expression, it is possible (and perhaps likely) that the set will be heterogeneous; that is, that different regulatory mechanisms are operating on different genes, but with similar results. Each mechanism may involve multiple TFs, and thus multiple TFBS. The danger in motif clustering and PWM construction is in combining unrelated motifs, a problem we have encountered repeatedly in practice. We speculate this may be due in part to the enormous flexibility of the (unrestricted) PWM model. Despite these potential pitfalls, some level of degeneracy is essential for improving sensitivity. An open question is whether one could sufficiently restrict PWM construction (e.g. with an appropriate prior) to provide some degeneracy while avoiding over-generalizing motifs. In the absence of such methods, we believe that the word-set model offers a safe and useful level of degeneracy that is resistant to overgeneralization. In our implementation, we elect not to further cluster the results, despite some redundancy. This is appropriate and sometimes useful to the specialist, but further post-processing would be desirable in a push-button tool targeted at novice users.

One might expect that the single-mutation word-set model would fail for motifs with more than one degenerate position, yet this does not appear to be a major problem in practice. For example, POWRS finds the correct motif for E2F and HNF1a, each of which has multiple degenerate positions (Table 2). We hypothesize this is because even for degenerate motifs, most occurrences are within one mutation of the consensus. Using the literature E2F motif as an example, the word-set model could capture three of the four main possibilities for the degenerate center bases (the consensus “GG” and variants “GC” and “CG”), and could thus match >75% of occurrences. While POWRS does fail to detect the highly degenerate Sox2 motif, none of the other methods fared any better. Since many of those methods are PWM-based, it seems unlikely that choice of motif model is the deciding factor.

Having adopted the word-set motif model, we find no need for an elaborate scoring function. We chose the binomial distribution over the hypergeometric because the former admits use of the binned enrichment correction described by Linhart et al. [3] and is somewhat faster; otherwise, there is no practical difference. (We have found the binned enrichment correction helpful in the past, but have not needed it since accounting for positional effects.) One important choice, however, is whether to count the number of sequences that contain a motif or the number of occurrences of a motif (irrespective of how many sequences they are spread over). We find the latter approach very susceptible to false positive enrichments, likely due to duplications and similar events within a small subset of the sequences.

Scoring based on the binomial (or hypergeometric) distribution has the nice property of balancing sample size against degree of enrichment in a simple but statistically principled way. A motif that matches 90% of the genes of interest and is weakly enriched may be more or less significant than a motif that matches 10% of those genes but is strongly enriched. Nonetheless, these extreme cases can be directly compared by their p-values. A small number of genes that achieve high statistical significance (i.e. strong enrichment) are particularly interesting for motif discovery in co-regulated sets of genes. Any collection of approximately “co-regulated” genes will likely contain a significant amount of heterogeneity in terms of expression pattern. Thus, it is implausible that all genes in the set would be controlled by exactly the same (set of) TFs. Finding that 10% of such a set (∼12 genes here) share a common regulatory motif would suggest that those genes may have some functional relationship that should be investigated further.

The utility of positional preferences in the discovery process varies by motif. Some TFBS appear to be relatively non-specific in their positioning (e.g. ETS1, ∼550 bp window). These motifs should be discoverable by any method when applied to proximal promoter sequences (e.g. -500 to -1 positions). On the other hand, some motifs are highly localized, such as the TATA box (25 bp window) and the telo box (100 bp window). For discovering these motifs, positioning information is very helpful, and restricting the sequence range *a priori* is difficult. Fortunately, POWRS can efficiently detect either type of motif distribution, even when presented with very long input sequences (e.g. −2000 to −1). Lastly, because the binomial test balances statistical power against specificity, we cannot say that ETS1 does not prefer a narrower range of positions, only that with the data in question, we lack the statistical power to narrow it further. The same statistical balancing act will necessarily exclude some number of biological relevant “far” TFBS that occur outside the ranges determined by POWRS.

During the development and validation of POWRS, several other algorithms focused on motif position have been published [17], [30]–[32]. For comparison, we report benchmark results for one of these (Dispom) in Table 1 [17]. In particular, Narang et al. [32] independently developed an approach quite similar to POWRS. Among the major differences, they employ a weighted combination of three separate scoring functions formulated in terms of entropy, while POWRS’ single score sidesteps their unresolved problem of choosing good weights. Additionally, Narang et al. [32] use a consensus sequence plus all variants instead of an optimized subset, use a Markov model for background, and do not identify strand preferences. Unfortunately, we were unable to generate meaningful benchmark results with the provided implementation. However, these authors all concur that incorporating position information improves their motif discovery algorithms.

Benchmarking algorithms is difficult; doing so fairly and informatively is extremely challenging. An exemplary evalution of drug docking algorithms by Warren et al. [33] discusses many potential pitfalls for the unwary. Such a comprehensive benchmark is well beyond the scope of this study; it could be argued no comparably rigorous comparative evaluation yet exists in the motif discovery literature. Thus, we do not assert that POWRS is superior to other algorithms on the basis of the data here, only that it is approximately comparable to the state of the art, and that it appears to provide some novel positional information. While experience with internal projects leads us to hypothesize that POWRS does offer some unique advantages, confirmation of that will require additional empirical data.

In the 18 cases examined, the top 3 programs performed essentially identically; and when successful, the correct motif is generally in the top two results. However, in three cases, no program was successful. This may mean that the motif is not enriched in the target sequences, but is only active there because of pairing with other factor(s) and/or structural features. Such motifs will require very different algorithms, if it is even possible to discover them computationally. Alternately, the data may simply be low quality. In the miRNA cases, the two “difficult” cases are paired with “easy” data sets for the same motif where many programs succeed. This strongly suggests data quality is the issue, underscoring the need for careful curation of benchmark data.

On the other hand, we see little value in benchmarks on synthetic data. Such data sets inevitably reflect the preconceptions of their creators, but they also often include blatantly unrealistic features, such as equal A/C/G/T content, zero-order Markov models, and exactly one instance of the true motif embedded in each input sequence. In our experience, even high-order Markov models do a poor job recapitulating the quirks of real sequences, which include long runs of repetitive and low-complexity sequence, sequence duplication (including transposons), nucleosome positioning and other structural features [34], [35], etc. Thus, one useful future direction for the field is to exploit these “quirks” to enhance discovery, as we have done here with motif position and strand preferences.

## Supporting Information

Table S1 List of the 118 constitutive highly-expressed Arabidopsis genes.
(XLS)

Table S2 Detailed results of POWRS motif searches with graphical representations of JASPAR PWMs.
(DOCX)
