# Supplementary material for: POWRS: Position-Sensitive Motif Discovery
Source: PLoS One. 2012 Jul 5;7(7):e40373. doi: 10.1371/journal.pone.0040373 (PMC3390389; doi:10.1371/journal.pone.0040373)
Supplement: Table S2 — Detailed results of POWRS motif searches with graphical representations of JASPAR PWMs. (DOCX) [file pone.0040373.s002.docx]

| Target | Ref. | Rank | Score | Motif | Start | End | Strands? |
| --- | --- | --- | --- | --- | --- | --- | --- |
| CREB | 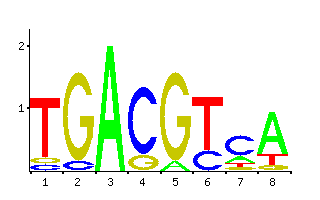 | 2 | 45.4 | [Act][Gat][Tg][Gc]ACG[Tac] | -400 | 0 | Both |
| E2F | 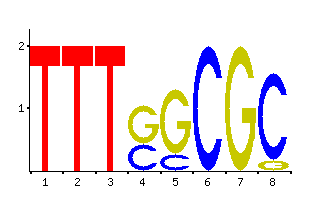 | 2 | 10.7 | TT[Ga][Gt]C[Ga]C[Gc] | -450 | -50 | Both |
| ETS1 | 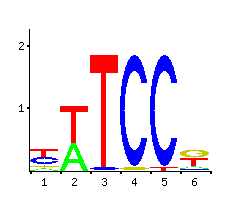 | 2 | 50.8 | [Cagt]A[Cagt]TTCCG | -550 | 0 | Both |
| HNF1a | 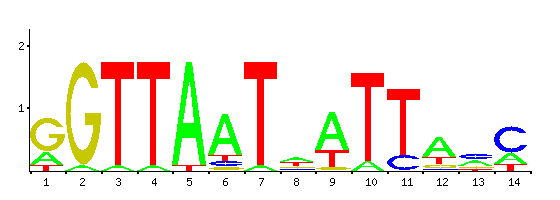 | 1 | 15.2 | TTA[Ac][Tc][Gac]A[Tcg] | -250 | 0 | Both |
| NFkB1 | 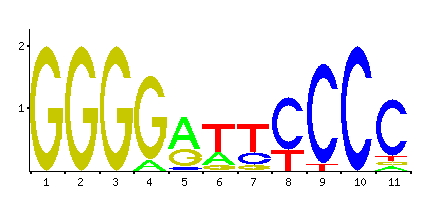 | 1 | 11.4 | G[Gt][Ag][At][At][Tac][Cat]C | -400 | 0 | Both |
| P53 | 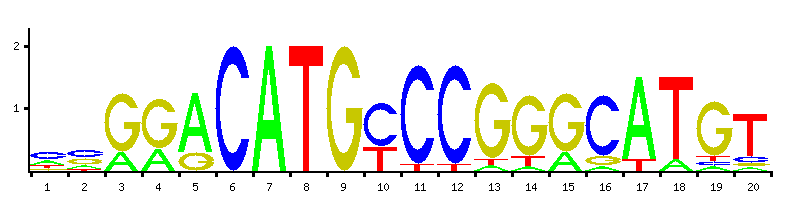 | - | - | - | - | - | - |
| Sox2 | 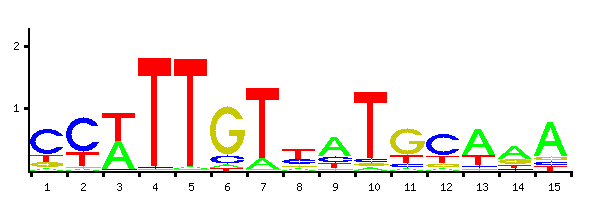 | - | - | - | - | - | - |
| SRF | 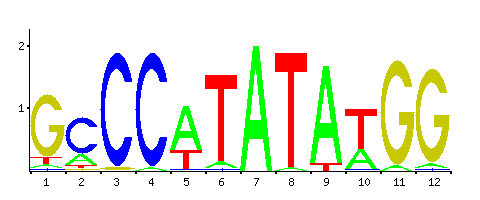 | 1 | 12.8 | C[Ca][At][Ta][Agt]T[At][Ta] | -300 | 0 | One |
| YY1 | 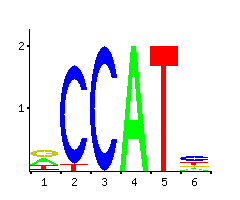 | 1 | 67.2 | GC[Cg]AT[Gact]T[Tc] | -350 | 0 | Both |
| let7 (B) | CTACCTCA | - | - | - | - | - | - |
| let7 (J) | CTACCTCA | 1 | 24.3 | [Ca]TA[Cag]CT[Cg][Ta] | 0 | 5000 | One |
| miR106b | GCACTTTA | 1 | 29.1 | GCACTTT[Act] | 0 | 4000 | One |
| miR124 | GTGCCTTA | 1 | 31.9 | [Gt]TGCCTT[Acgt] | 0 | 5000 | One |
| miR16 | TGCTGCTA | 1 | 31.3 | [Tac]GCTGCT[Agt] | 0 | 3500 | One |
| miR1 | ACATTCCA | 1 | 23.8 | [Agt]CATTCC[Agt] | 0 | 2000 | One |
| miR34 (C) | CACTGCCT | - | - | - | - | - | - |
| miR34 (H) | CACTGCCT | 1 | 45.9 | [Cagt]AC[Tag]GCC[Tag] | 0 | 2000 | One |
| miR373 | AGCACTTC | 1 | 18.4 | [Tac]A[Ag]GCACT | 0 | 1000 | One |
| telo box | AAACCCTAGC | 1 | 28.0 | [Ag]C[Ct]C[Ta][At][Gat][Tacg] | -75 | +25 | Both |
| Site II | AAGGCCCAWT | 2 | 23.4 | [Agt][Gat][Ga]CC[Cg]A[Acgt] | -150 | -25 | One |
| TATA box | TCTATAAAA | 3 | 17.2 | [Tacg][Ca][Tg]ATAA[Ag] | -50 | -25 | One |

Table S2: Detailed results of POWRS motif searches

“Target” refers to the data sets from [[3](#_ENREF_3)]. Reference motifs are PWMs from JASPAR (human TFs), seed sequences from miRbase (human miRNAs), or manual consensus sequences (Arabidopsis). Motifs are represented with the primary bases in uppercase and the variant bases in lowercase, with degenerate positions grouped in square brackets. Matching words are those that use at most one variant base, so [Tac]GCTGCT[Agt] = {TGCTGCTA, aGCTGCTA, cGCTGCTA, TGCTGCTg, TGCTGCTt}.
